# Multi-drug therapy for epilepsy influenced bispectral index after a bolus propofol administration without affecting propofol’s pharmacokinetics: a prospective cohort study

**DOI:** 10.1038/s41598-020-58460-2

**Published:** 2020-01-31

**Authors:** Matsuri Kodama, Hitoshi Higuchi, Minako Ishii-Maruhama, Mai Nakano, Yuka Honda-Wakasugi, Shigeru Maeda, Takuya Miyawaki

**Affiliations:** 1 Department of Dental Anesthesiology and Special Care Dentistry, Okayama University Graduate School of Medicine, Dentistry and Pharmaceutical Sciences, Okayama, Japan; 2 Department of Dental Anesthesiology, Okayama University Hospital, Okayama, Japan

**Keywords:** Drug regulation, Phase IV trials

## Abstract

Some previous studies have indicated that valproate (VPA) might change the pharmacokinetics and enhance the effects of propofol. We evaluated whether clinical VPA therapy affected the propofol blood level, the protein-unbound free propofol level, and/or the anesthetic effects of propofol in the clinical setting. The subjects were divided into the control group (not medicated with antiepileptics), the mono-VPA group (medicated with VPA alone), and the poly-VPA group (medicated with VPA, other antiepileptics, and/or psychoactive drugs). General anesthesia was induced via the administration of a single bolus of propofol and a remifentanil infusion, and when the bispectral index (BIS) exceeded 60 sevoflurane was started. There were no significant differences in the total blood propofol level at 5, 10, 15, and 20 min or the protein-unbound free propofol level at 5 min after the intravenous administration of propofol between the 3 groups. However, the minimum BIS was significantly lower and the time until the BIS exceeded 60 was significantly longer in the poly-VPA group. In the multivariate regression analysis, belonging to the poly-VPA group was found to be independently associated with the minimum BIS value and the time until the BIS exceeded 60. Clinical VPA therapy did not influence the pharmacokinetics of propofol. However, multi-drug therapy involving VPA might enhance the anesthetic effects of propofol.

## Introduction

Anesthesia management allows patients to undergo otherwise unbearable operations involving severe pain and marked vital reactions. In addition, in children and uncooperative adults, anesthetic management can expedite procedures, such as dental treatment, that are not particularly uncomfortable, but require the patient not to move. In particular, in dental treatment for patients with intellectual disabilities (ID), anesthesia management is used for behavioral control. Propofol is the most commonly used intravenous anesthetic for achieving such behavioral control because the depth of anesthesia induced by propofol can be controlled to a greater degree than that induced by other anesthetics, and so propofol is widely used for sedation and inducing general anesthesia during dental treatment for patients with ID.

Many people with ID are complicated with epilepsy and receive antiepileptic therapy. A recent systematic review estimated the prevalence of epilepsy to be 22.2% among people with ID^1^. Valproate (VPA) is an antiepileptic drug (AED) with a broad therapeutic spectrum and so has become the most widely used AED worldwide^2^. It is also the most commonly used AED among our dental patients with ID. In our research, we have so far focused on the drug interactions between propofol and VPA, and we have previously reported that oral VPA reduced the propofol dose required for sedation^3^. Similar findings were reported in another study^4^, which suggested that drug interactions occur between propofol and VPA. We generated the following hypotheses regarding the mechanism responsible for such drug interactions. Our first hypothesis is that VPA increases the proportion of protein-unbound free propofol in the blood during anesthesia. There is a possibility that VPA displaces protein-bound propofol because high proportions of both propofol and VPA bind to blood proteins (propofol: 97 to 99%^5,6^, VPA: 93%^7^). This would lead to a significant increase in the level of active propofol, which can act on the γ -aminobutyric acid (GABA)_A_ receptor, in the central nervous system (CNS). Regarding this hypothesis, we have already shown that VPA increased the proportion of protein-unbound free propofol in an *in vitro* study^8^. Our second hypothesis is that VPA inhibits propofol-metabolizing enzymes. In previous *in vitro* studies, it was demonstrated that VPA inhibited propofol-metabolizing enzymes, such as cytochrome P-450 (CYP) 2C9^9^ and UDP-glucuronosyltransferase (UGT) 1A9^10^. This suggests that VPA might inhibit propofol metabolism and increase the level of propofol in the blood. Our third hypothesis is that propofol and VPA have synergistic effects at propofol’s action site in the CNS. Regarding the mechanism responsible for the effects of VPA, it is considered that VPA increase the levels of GABA in the CNS by inhibiting GABA transaminase^11^. This process might enhance the activity of propofol, which is mostly mediated via the upregulation of GABA-induced chloride currents. However, the abovementioned hypotheses are based on the findings of *in vitro* studies and have not been verified in clinical patients. The aim of this study was to verify our hypotheses in clinical patients. To do this, we examined the differences in the propofol blood level, the protein-unbound free propofol level, and the bispectral index (BIS) seen after the administration of propofol between patients that were and were not treated with VPA.

## Materials and Methods

The present study was approved by the ethics committee of Okayama University Graduate School of Medicine, Dentistry, and Pharmaceutical Sciences (approval number: 1966, approved on February 28, 2012), and it was registered in the University Hospital Medical Information Network (UMIN) Clinical Trials Registry (UMIN000008711). We have read the Helsinki Declaration and have followed its guidelines in this study.

### Patient eligibility

Candidates were selected from patients who underwent dental treatment or oral surgery under general anesthesia at Okayama University Hospital between June 2012 and June 2017. Patients who agreed to take part in this study, met the inclusion criteria, and did not meet the exclusion criteria were selected. Written informed consent was obtained from each subject, or the legal guardians of the subject if the patient had an ID that would make it difficult for them to provide informed consent. The inclusion criteria were as follows: (1) being ≥16 years old; (2) having an American Society of Anesthesiologists (ASA) physical status of 1 or 2; and (3) not taking medication or VPA-containing medication. The exclusion criteria were as follows: (1) requiring pretreatment, such as oral midazolam and/or sevoflurane inhalation, prior to venous catheter insertion; (2) being medicated with other AED without VPA; and (3) being contraindicated for the use of propofol, remifentanil, sevoflurane, or rocuronium. The patients were divided into the control group, who were not given any medication; the mono-VPA group, who were medicated with VPA alone; and the poly-VPA group, who medicated with VPA and other AED and/or psychoactive drugs.

### General anesthesia technique

General anesthesia was induced using the same procedure in all patients. The monitoring performed during the procedure involved non-invasive blood pressure measurement; electrocardiography; and the measurement of oxygen saturation (SpO_2_; with a pulse oximeter), end-tidal CO_2_ (EtCO_2_), and the BIS. After inserting a venous catheter, the intravenous administration of remifentanil was infused at 0.25 μg/kg/min (Terumo TE-351, Terumo, Tokyo, Japan) during 2 min, then 2 mg/kg propofol was intravenously administered at 1200 mL/h using an infusion pump (Graseby 3500, Graseby Medical Ltd., Watford, United Kingdom). After the patient became unconscious, a venous catheter for blood sampling was placed in the opposite arm. Then, 0.6 mg/kg of rocuronium was intravenously administered for tracheal intubation. After the administration of propofol, no additional anesthetics were used while the patient’s BIS was <60. Then, the administration of 1.5–3% sevoflurane was started when the patient’s BIS increased to ≥60. Anesthesia was freely maintained using sevoflurane and remifentanil at the anesthesiologist’s discretion after sevoflurane started.

### Outcome measures

The blood samples used to measure propofol levels were collected at 5, 10, 15, and 20 min after the administration of propofol, and the protein-unbound free propofol concentration was measured at 5 min after the administration of propofol. To evaluate the effect of propofol-induced anesthesia, the BIS before the administration of propofol, the minimum BIS before the initial administration of sevoflurane, and the time until the BIS reached ≥60 were determined. In addition, the VPA blood concentrations of the patients in the mono-VPA and poly-VPA groups were measured by the LSI Medience Corporation (Tokyo, Japan). Laboratory data, such as the patient’s serum levels of alanine aminotransferase (ALT), aspartate aminotransferase (AST), albumin (Alb), etc., were collected via preoperative blood examinations. In this study, patients with abnormal laboratory ALT, AST, and/or gamma-glutamyltransferase (γ-GT) values were suspected to have liver dysfunction. Similarly, patients with abnormal laboratory for blood urea nitrogen (BUN) or creatinine (Cr) values or abnormal estimated glomerular filtration rates (eGFR) were suspected to have renal dysfunction. Abnormal values were defined based on the Common Reference Intervals of the Japan Society of Clinical Chemistry^12^ and the Evidence-based Clinical Practice Guidelines for CKD of the Japanese Society of Nephrology.

### Measurement of propofol levels

The blood levels of propofol and the protein-unbound free propofol level were measured as described in our previous study^8^. Serum was collected from the blood samples by centrifuging the blood samples at 1,400 G for 10 min. To measure the protein-unbound propofol level, protein-free serum was extracted using centrifugal filter units (Centrifree®; Merck Millipore, Bedford, USA) by centrifuging the samples at 2,000 G for 30 min. For preparation, thymol (Nacalai Tesque, Inc., Kyoto, Japan), as an internal standard (IS); 0.1 M potassium dihydrogen phosphate solution (Merck KGaA, Darmstadt, Germany); and heptane solution (Sigma-Aldrich, Co., MO, USA) were added to each sample. After the resultant mixture was centrifuged at 13,000 G for 3 min, the propofol concentration of the supernatant was measured with a high-performance liquid chromatography (HPLC) system (Shimadzu Co., Tokyo, Japan).

The peak area ratio of propofol to the IS was obtained from the HPLC chromatogram at each control concentration, and calibration curves for the blood propofol level (0.156, 0.312, 0.625, 1.25, and 2.5 μg/mL) and the protein-unbound free propofol level (0.005, 0.01, 0.02, and 0.04 μg/mL) were constructed.

### Statistical analysis

The results are presented as mean ± standard deviation values. Continuous variables were analyzed via one-way analysis of variance (ANOVA) with Tukey’s multiple comparisons test, and categorical variables were analyzed using the chi-square test or Fisher’s exact test. Mixed-effect analysis was employed for comparisons of propofol blood levels. In the analysis of BIS values, we employed an analysis of covariance (ANCOVA) model, in which the presence of ID was employed as a covariate, because our previous study indicated that the presence/absence of ID influenced BIS values during propofol anesthesia^13^. Multiple linear regression analyses were performed to control for potential confounding variables and to identify factors that are independently associated with BIS values. The group and eight factors (age, sex, BMI, ID, liver dysfunction, the Alb concentration, and the blood levels of propofol and protein-unbound free propofol), which were considered to potentially affect BIS values when propofol was administered^8,13–16^, were included as potential predictors of BIS values. ANOVA, the chi-square test, Fisher’s exact test and Mixed-effect analysis were performed using the GraphPad Prism statistical software (GraphPad Prism, ver. 8; GraphPad Software, Inc., La Jolla, CA, USA). ANCOVA was performed with SAS ver.9.4 (SAS Institute, Inc., Cary, NC), and the multivariate analysis was carried out with JMP ver.14.3.0 (SAS Institute, Inc., Cary, NC). P-values of <0.05 were regarded as statistically significant.

## Results

In total, 12, 10, and 11 subjects were included in the control group, mono-VPA group, and poly-VPA group, respectively. The demographic and clinical characteristics of the subjects in each group are summarized in Table 1. There were no significant differences in terms of sex, age, height, body weight, or body mass index among the groups. The levels of protein, and Alb also did not differ among the groups. There were two, two, and three patients with suspected liver dysfunction in the control group, mono-VPA group, and poly-VPA group, respectively. There were no patients with suspected renal dysfunction in this study. However, the mono-VPA and poly-VPA groups included more patients with ID than the control group. Table 2 shows the details of the medications administered in the mono-VPA and poly-VPA groups. Carbamazepine and phenytoin were the drugs that were most frequently added to VPA therapy, and the combination of 2 or 3 agents, including VPA, was common in the poly-VPA group. There was no difference in the VPA concentration between the mono-VPA and poly-VPA groups.

**Table 1:** Subjects’ demographic characteristics.

	Control	Mono	Poly	P-value
Sex (male/female)	8/4	4/6	9/2	0.133
Age (years)	29.5 ± 6.5	30.2 ± 10.1	28.5 ± 9.1	0.90
Height (cm)	163.0 ± 8.8	157.4 ± 7.9	164.7 ± 14.1	0.28
Weight (kg)	56.7 ± 14.0	57.2 ± 19.6	61.3 ± 16.1	0.78
BMI (kg/m^2^)	21.1 ± 3.9	22.9 ± 6.8	22.2 ± 3.3	0.69
Intellectual disability (Yes/no) (n)	4/8	8/2^*^	11/0^**^	
TP (g/dL)	7.47 ± 0.51	7.21 ± 0.48	7.16 ± 0.56	0.33
Alb (g/dL)	4.63 ± 0.23	4.37 ± 0.25	4.35 ± 0.45	0.075
Suspected liver dysfunction (Yes/no) (n)	2/10	2/8	3/8	
Suspected renal dysfunction (Yes/no) (n)	0/12	0/10	0/11	

Data are presented as mean ± SD values.

*P < 0.05 (vs. control group, Fisher’s exact test).

**P < 0.01 (vs. control group, Fisher’s exact test).

Control: control group, Mono: mono-VPA group, Poly: poly-VPA group.

BMI: body mass index, TP: total protein, Alb: albumin.

See text for definitions of suspected liver and renal dysfunction.

**Table 2:** Breakdown of the medicines used in the mono-VPA and poly-VPA groups.

Group	Mono	Poly
**Number of medications**		
1	10 [100]	
2	—	5 [45]
3	—	4 [36]
4	—	1 [9]
7	—	1 [9]
**Antiepileptic drugs**		
Valproate	10 [100]	11 [100]
Valproate blood level (µg/mL)*	53.9 ± 15.8	65.7 ± 21.4
Carbamazepine	—	4 [36]
Phenytoin		3 [27]
Phenobarbital	—	1 [9]
Clobazam	—	1 [9]
Clonazepam	—	1 [9]
Levetiracetam	—	1 [9]
Zonisamide	—	1 [9]
**Antipsychotic drugs**		
Risperidone		2 [18]
Quetiapine	—	1 [9]
Biperiden	—	1 [9]
Pimozide	—	1 [9]
Paliperidone	—	1 [9]
Haloperidol	—	1 [9]
Aripiprazole		1 [9]
**Antidepressant drugs**		
Etizolam	—	1 [9]
Fluvoxamine	—	1 [9]

Data are presented as absolute [percentage] or mean ± SD* values.

Mono: mono-VPA group, Poly: poly-VPA group.

**Figure 1:**
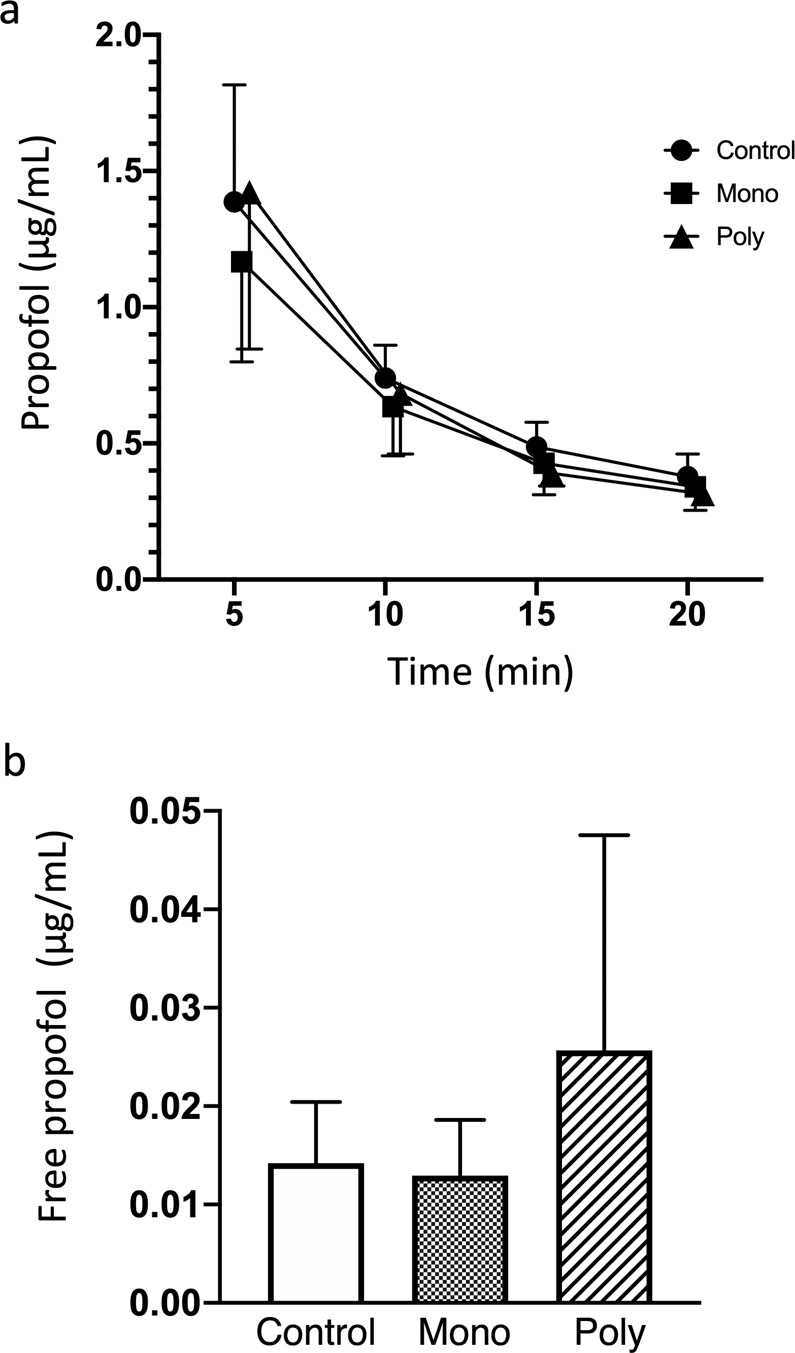
The effects of mono-VPA and poly-VPA therapy on the pharmacokinetics of propofol. The graphs show the blood levels of propofol at 5, 10, 15, and 20 min after the administration of propofol (**a**) and the concentration of protein-unbound free propofol at 5 min after the administration of propofol. (**b**) There were no significant differences among the 3 groups. However, the protein-unbound free propofol level tended to be higher in the poly-VPA group. Mixed-effect analysis was employed for the analysis of propofol blood levels, and one-way ANOVA with Tukey’s multiple comparisons test were used to analyze the concentration of protein-unbound free propofol. Data are presented as mean ± standard deviation (SD) values. Control: control group, Mono: mono-VPA group, Poly: poly-VPA group.

The blood levels of propofol at each point after the administration of the drug and the concentration of protein-unbound free propofol at 5 min after the administration of the drug are shown in Fig. 1. There were no significant intergroup differences in the total blood propofol level at any point. The concentration of protein-unbound free propofol tended to be higher in the poly-VPA group, but the difference was not significant. This indicates that VPA therapy did not directly influence the pharmacokinetics of propofol after the administration of a single dose of the drug.

**Figure 2:**
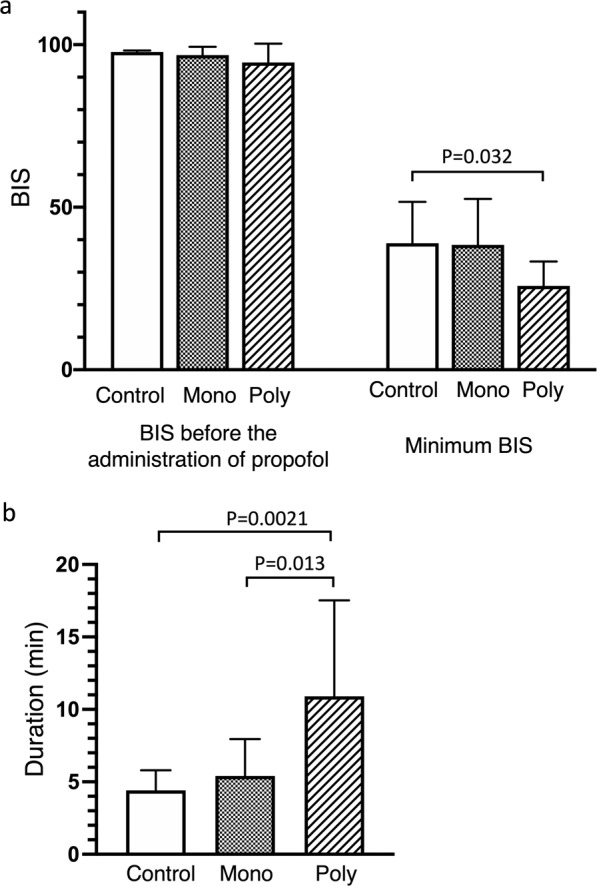
The effects of mono-VPA and poly-VPA therapy on the BIS values seen after the administration of propofol. In the poly-VPA group, the minimum BIS (**a**) was significantly lower, and BIS of <60 were maintained for significantly longer (**b**) than in the other groups. The statistical analyses were performed via one-way ANOVA with Tukey’s multiple comparisons test. The data are presented as mean ± SD values. Control: control group, Mono: mono-VPA group, Poly: poly-VPA group.

Regarding the BIS, the BIS before the administration of propofol (control group: 97.8 ± 0.5, mono-VPA group: 96.8 ± 2.6, poly- VPA group: 94.5 ± 5.8) was not difference among the groups. The minimum BIS before the initial administration of sevoflurane (control group: 38.9 ± 12.8, mono-VPA group: 38.4 ± 14.1, poly-VPA group: 25.8 ± 7.5) was significantly lower in the poly-VPA group than in the control group according to ANOVA (p = 0.032) (Fig. 2), but this difference was not found to be significant when ANCOVA was performed using the presence of ID as a covariate (p = 0.057). However, a significant difference was detected between the mono-VPA and poly-VPA groups according to ANCOVA (p = 0.049). The time until the BIS exceeded 60 (control group: 4.4 ± 1.4 min, mono-VPA group: 5.4 ± 2.5 min, poly-VPA group: 10.9 ± 6.6 min) was also significantly longer in the poly-VPA group than in the control and mono-VPA groups by ANOVA (vs. the control group: p = 0.0021, vs. mono-VPA group: p = 0.013) (Fig. 2), and the significance of these differences was not affected by performing ANCOVA using the presence of ID as a covariate (vs. the control group: p = 0.006, vs. mono-VPA group: p = 0.012).

Finally, multivariate linear regression models were constructed using the minimum BIS value or the time until the BIS exceeded 60 as dependent variables. The estimated coefficient, standard error, and P-values for these variables are presented in Tables 3 and 4. In the multivariate regression analysis, belonging to the poly-VPA group was shown to be independently associated with the minimum BIS value or the time until the BIS exceeded 60, and was also found to significantly affect the BIS value. These findings indicate that polytherapy involving VPA enhanced the effects of propofol in clinical patients.

**Table 3:** Multiple regression analysis of predictors of the minimum BIS value.

Variable	Estimated coefficient	Standard error	P-value
Intercept	71.815	39.940	0.088
Group (poly-VPA)	9.933	3.249	0.007
Age	−0.229	0.303	0.460
Sex (female)	−4.828	2.880	0.110
BMI	−0.692	0.615	0.274
Non-ID	−2.058	2.972	0.497
Non-liver dysfunction	−4.314	3.023	0.170
Alb	−2.707	7.666	0.728
Propofol level	−7.038	8.830	0.435
Free propofol level	205.673	285.108	0.480

R^2^ = 0.455.

BMI: body mass index, ID: intellectual disability, Alb: albumin.

See text for the definition of suspected liver dysfunction.

**Table 4:** Multiple regression analysis of predictors of the time until the BIS exceeded 60.

Variable	Estimated coefficient	Standard error	P-value
Intercept	9.018	7.340	0.234
Group (poly-VPA)	−1.951	0.597	0.004
Age	0.074	0.056	0.198
Sex (female)	−0.044	0.529	0.934
BMI	0.177	0.113	0.134
Non-ID	0.311	0.546	0.575
Non-liver dysfunction	−0.301	0.556	0.594
Alb	−1.553	1.409	0.284
Propofol level	−1.297	1.623	0.434
Free propofol level	34.337	52.394	0.520

R^2^ = 0.620.

BMI: body mass index, ID: intellectual disability, Alb: albumin.

See text for the definition of suspected liver dysfunction.

## Discussion

In this study, to evaluate the effects of VPA therapy on the pharmacokinetics of propofol, we compared the total and protein-unbound free propofol levels seen after the administration of a single bolus of propofol among patients treated without VPA, patients treated with mono-VPA therapy, and patients treated with poly-VPA therapy. As a result, we found that the BIS was significantly lower and a BIS of <60 was maintained for significantly longer in the poly-VPA group. However, there were no significant differences in the total or protein-unbound free propofol level between the 3 groups, and the pharmacokinetics of propofol were not altered by VPA therapy.

Drug-drug interactions involving AED are common in clinical practice. An estimated 6% of cases of AED intoxication are related to drug-drug interactions^17^. In general, antiepileptic monotherapy has been found to be effective in 60% to 70% of newly diagnosed epileptic patients, while up to 50% of patients in whom antiepileptic treatment initially fails can be managed by switching them to alternative antiepileptics^17^. Furthermore, epileptic patients who do not respond to monotherapy are typically administered a combination of antiepileptics to improve their seizure control^17^. However, polytherapy is expected to increase the risk of drug-drug interactions.

It is widely known that AED induce or inhibit CYP activity and influence drug metabolism. Most AED induce CYP activity^18^, but it has also been shown that VPA inhibits CYP2C9, CYP2C19, and CYP3A4 activity and has the potential to inhibit drug metabolism, resulting in a number of interactions involving elevated plasma concentrations of concomitantly administered drugs^2^.

Ninety percent of administered propofol is metabolized in the liver, and the resultant metabolites are excreted in urine. Both phase I (hydroxylation) and phase II (glucuronidation) processes contribute to propofol metabolism. Seventy percent of the drug is converted to propofol glucuronide by uridine diphosphate UGT1A9, while CYP2B6 and, to a lesser extent, CYP2C9 are responsible for the hydroxylation of propofol to 1- and 4-quinol metabolites^19^. *In vitro* studies demonstrated that VPA inhibited CYP2C9^9^ and UDP-glucuronosyltransferase (UGT) 1A9^10^, and these findings led to the hypothesis that VPA inhibits propofol metabolism and increases the blood level of propofol. However, it has been suggested that VPA only weakly inhibits CYP2C9^17^, and the inhibition of UGT1A9 by VPA was detected at a high VPA concentration. Therefore, VPA might not influence the blood level of propofol in the clinical setting, as indicated by our results. In addition, our study suggested that other AED and psychoactive drugs had no influence on the propofol level because the total propofol level did not differ between the mono-VPA and poly-VPA groups.

Another drug-drug interaction between VPA and propofol that needs considering is that VPA can displace serum protein-bound propofol, which increases the proportion of protein-unbound free propofol. A high proportion of propofol (97 to 99%) binds to serum proteins, and it is considered that the protein-unbound free propofol concentration is important for the pharmacodynamics of propofol because free propofol has knock-on effects on propofol’s target proteins, such as the GABA_A_ receptor. Regarding this matter, in a previous *in vitro* study we suggested that VPA might affect the binding of propofol to serum proteins and increase the proportion of protein-unbound free propofol in serum^8^. However, these results were obtained at a low Alb concentration (2.5 g/dL) and a VPA concentration of ≥100 μg/mL. In the current study, the VPA concentration was 64.0 ± 21.4 μg/mL in the mono-VPA group and 58.0 ± 15.3 μg/mL in the poly-VPA group, and the VPA concentrations of both groups were <100 μg/mL. Moreover, the Alb level was >4.0 g/dL in all groups. Therefore, the VPA and Alb levels of our subjects differed from those found in our previous *in vitro* study, and VPA did not influence the protein-unbound free propofol concentration. However, the protein-unbound free propofol level tended to be higher in the poly-VPA group than in the other groups although the differences were not significant. The protein-bound proportions of carbamazepine (75%), phenytoin (~90%)^20^, and risperidone (90%)^21^, which are the other main drugs that are combined with VPA, are also high, and it is possible that the combined use of these drugs might have influenced the protein binding of propofol in the present study.

Another conceivable mechanism that might underlie the drug-drug interactions between propofol and VPA is synergistic effects at propofol’s site of action in the CNS. The hypnotic action of propofol is mostly mediated via the enhancement of GABA-induced chloride currents through the binding of the drug to the β-subunit of the GABA_A_ receptor^22^. VPA inhibits succinate semialdehyde dehydrogenase and GABA transaminase, and consequently, the brain levels of GABA tend to increase^11^. This might lead to synergistic effects between propofol and VPA. In our study, there was no difference in the minimum BIS or the time for which the BIS remained <60 between the control group and the mono-VPA group. This indicates that VPA monotherapy had no synergistic effects when combined with propofol. However, in the poly-VPA group, the BIS was significantly lower and BIS of <60 were maintained for significantly longer. In the poly-VPA group, other AED (such as carbamazepine and phenytoin), antipsychotics, and antidepressants were used as additional drugs (Table 2). The molecular targets of AED are Na^+^ channels, Ca^2+^ channels, the GABA_A_ receptor, etc^23^, and these drugs inhibit abnormal neuronal excitation through these targets. Thus, it can be inferred that these effects might also enhance the anesthetic effects of propofol. Moreover, the main mechanism of action of antipsychotics involves antagonism of the dopamine D2 receptor. A recent study showed that droperidol, a dopamine D2 receptor antagonist, enhanced sevoflurane-induced anesthesia^24^, so some antipsychotics, which have antagonistic effects on the D2 receptor, also enhance anesthetic effects. Our results demonstrated that there were no differences in the blood concentration or protein-unbound free concentration of propofol among the 3 groups; therefore, it is presumed that synergistic effects at propofol’s site of action in the CNS affected the BIS of propofol, and so the BIS of propofol was significantly lower and BIS of <60 were maintained for significantly longer in the poly-VPA group.

However, in our previous study^3^, the required doses of propofol were significantly lower in the mono-VPA group than in the control group, and mono-VPA therapy altered the effects of propofol, unlike in the present study. So, why were there discrepancies between the results of our two studies? The reason for this is unclear. However, midazolam was used in combination with propofol for sedation in the previous study, and midazolam might have similar effects to poly-AED therapy. Midazolam was also suggested to have such effects in our previous study. This retrospective cohort study suggested that the amount of intravenous midazolam administered is an independent determinant of the time to recovery from general anesthesia^25^. In this way, there is a possibility that the use of multiple CNS depressants affects the BIS during general anesthesia or sedation. However, we were not able to directly examine this hypothesis in the current study.

This study had a few limitations that need to be mentioned. First, there is the influence of epilepsy on the BIS. The BIS is derived from electroencephalography (EEG) and has been reported to be useful for measuring the hypnotic component of anesthesia. No detailed method for calculating the BIS has yet been published. Epilepsy attacks obviously induce EEG changes; however, there have been few reports about the abnormal BIS values observed in patients with epilepsy. In addition, there have reports about both increases^26,27^ and decreases in the BIS during seizures^28–30^, so the changes in the BIS seen during seizures remain unclear. In our study, most patients had well-controlled epilepsy, but some patients suffered regular small seizures. Despite this, there were no differences in the BIS seen before the induction of general anesthesia among the 3 groups, and no sudden changes in the BIS value, which were reported during epileptic seizures in previous studies^28,29^, were noted in the present study. Therefore, we consider that epilepsy did not directly affect the BIS in our study. The second limitation is the influence of ID on the BIS. We have reported that patients with ID exhibited significantly lower BIS during general anesthesia than patients without ID, and patients with ID took longer to emerge from anesthesia than patients without ID^13^. In the current study, the control group included 4 patients with ID (33%), the mono-VPA group included 8 patients with ID (80%), and the poly-VPA group included 11 patients with ID (100%); i.e., more patients with ID were included in the mono-VPA and poly-VPA groups than in the control group. It is possible that such population biases influenced our results. Therefore, we added an ANCOVA model, in which the presence of ID was employed as a covariate, to adjust for imbalances in this variable. Moreover, we analyzed our results using multiple linear regression to control for potential confounding variables. As a consequence, both analyses indicated that the use of multiple CNS depressants, including VPA, significantly affected the BIS value. The third limitation is that this study could not clarify which factors in the poly-VPA group, such as the number of AED, a certain kind of AED, or the amount of AED, etc., influenced our results. As the combinations of AED used in the poly-VPA group were not determined, it was very hard to evaluate these matters. The fourth limitation is that our sample size was small. This study was carried out at a single center, and it was hard to find patients who met the inclusion criteria, did not meet the exclusion criteria, and agreed to participate in our study. However, in the analysis of the time taken to reach a BIS of ≥60 a power (1-β) value of 0.83 was achieved, which was sufficient. We will plan future studies in consideration of the limitations of this study.

In conclusion, based on our previous studies, we hypothesized that VPA therapy would alter the pharmacokinetics of propofol and enhance its effects, and we evaluated our hypothesis in this study. As a result, we found that VPA therapy did not influence the pharmacokinetics of propofol. However, in poly-VPA therapy; i.e., combination therapy involving VPA and other AED or antipsychotics, significantly lower BIS were seen, and BIS of <60 were maintained for significantly longer. The findings of the present study suggest that using multiple CNS depressants, such as AED or antipsychotics, might decrease the BIS and maintain low BIS values for longer due to synergistic effects at the site of action of propofol in the CNS. We must pay close attention to patients who undergo multi-drug therapy for epilepsy during the management of anesthesia because the BIS might be affected by these drugs.

## Data Availability

The datasets generated during and/or analyzed during the current study are available from the corresponding author on reasonable request.
